# Pandemic-Proofing: Intercepting Zoonotic Spillover Events

**DOI:** 10.3390/pathogens13121067

**Published:** 2024-12-03

**Authors:** Bharti Bhatia, Sudipta Sonar, Seema Khan, Jayanta Bhattacharya

**Affiliations:** 1 Molecular and Translational Virology, Centre for Virus Research, Vaccines and Therapeutics, Translational Health Science and Technology Institute, NCR Biotech Science Cluster, Faridabad 121001, India; 2 Antibody Translational Research Program, Translational Health Science and Technology Institute, NCR Biotech Science Cluster, Faridabad 121001, India

**Keywords:** root cause, zoonosis, prevention, historical spillovers, one health, surveillance and healthcare

## Abstract

Zoonotic spillover events pose a significant and growing threat to global health. By focusing on preventing these cross-species transmissions, we can significantly mitigate pandemic risks. This review aims to analyze the mechanisms of zoonotic spillover events, identify key risk factors, and propose evidence-based prevention strategies to reduce future pandemic threats. Through a comprehensive literature review and analysis of major databases including PubMed, Web of Science, and Scopus from 1960–2024, we examined documented spillover events, their outcomes, and intervention strategies. This article emphasizes that targeting the root cause—the spillover event itself—is key to averting future pandemics. By analyzing historical and contemporary outbreaks, we extract crucial insights into the dynamics of zoonotic transmission. Factors underlying these events include increased human–animal contact due to habitat encroachment, agricultural intensification, and wildlife trade. Climate change, global travel, and inadequate healthcare infrastructure exacerbate risks. The diversity of potential viral reservoirs and rapid viral evolution present major challenges for prediction and prevention. Solutions include enhancing surveillance of wildlife populations, improving biosecurity measures, investing in diagnostic capabilities, and promoting sustainable wildlife management. A “One Health” approach integrating human, animal, and environmental health is crucial. Predictive modelling, international cooperation, and public education are key strategies. Developing pre-exposure prophylactics and post-exposure treatments is essential for mitigating outbreaks. While obstacles remain, advances in genomics and ecological modelling offer hope. A proactive, comprehensive approach addressing the root causes of spillover events is vital for safeguarding global health against future pandemics.

## 1. Introduction

The adage “prevention is better than cure” holds true not only for individual health but also for pandemics. We must shift our focus from reactive crisis management towards proactive prevention to avoid the devastating human and economic cost of outbreaks. The recent havoc caused by Severe Acute Respiratory Syndrome (SARS) Corona virus exemplifies this risk [1]. We may be facing a future where COVID-19 is ever present due to unsuccessful containment efforts [2].

A brief look at the history of pandemics (Table 1) reveals that the primary cause is the transmission of pathogens through ‘species crossover’ or ‘zoonotic spillover’ [3]. The Black Death, a devastating epidemic in 14th century Europe, is thought to have started in rodents and spread to humans via fleas [4]. The 1918 influenza pandemic, known as the Spanish Flu, likely crossed from birds to humans, resulting in millions of deaths globally [5]. Human immunodeficiency virus/Acquired immunodeficiency syndrome (HIV/AIDS) emerged from a strain of Simian immunodeficiency virus (SIV) in chimpanzees [6]; in the 21st century, series of deadly disease outbreaks, such as avian influenza A (H5N1) [7], influenza A (H1N1) [8], and most recently, COVID-19, happened; the severe acute respiratory syndrome coronavirus 2 (SARS-CoV-2), a member of the *Coronaviridae* family [9].

Other notable zoonoses with pandemic potential include Middle East respiratory syndrome (MERS) [28], Ebola virus disease [29], Zika virus disease [30], Nipah virus infections [31], West Nile fever [32,33], Rift Valley Fever [34], Rabies [35], and Mpox [36]. The H5N1 influenza virus, known for its avian origins, has recently been detected circulating in cattle, raising concerns about its potential for further adaptation and spread [37].

Our actions, such as encroaching on wildlife habitats, unsustainable hunting practices, and intensive farming, have brought us dangerously close to animal reservoirs of diseases. This creates a perfect breeding ground for pathogens to jump species. Understanding the factors underlying the zoonotic spillover is instrumental in developing methods to detect and control the pathogen’s spread.

Proactive surveillance and monitoring of wildlife populations can aid in early detection of novel pathogens or unusual disease patterns [38,39]. Enhancing biosafety and biosecurity protocols in animal agriculture operations can mitigate the zoonotic transmission risks posed by these concentrated reservoirs of potential pathogens. Furthermore, stringent regulations governing the trade and consumption of wildlife can contribute to diminishing the likelihood of zoonotic spillover events from sylvatic reservoirs into human populations. Such comprehensive approaches targeting the interfaces between animal reservoirs and human exposure are pivotal for pre-empting the emergence of zoonoses with pandemic potential [40,41] and are crucial for long-term prevention. Investment in research, development of diagnostic tools, and the discovery of effective therapeutics and vaccines can strengthen our preparedness and response capabilities against emerging zoonotic threats.

Once a spillover event is detected, rapid response teams can be deployed to identify the source, trace contacts, and implement containment measures. Effective communication strategies play a vital role in raising public awareness, promoting behavior change, and disseminating guidance on prevention and treatment [42]. Collaborative efforts between human and animal health sectors, known as the One Health approach, are essential for comprehensive surveillance, risk assessment, and coordinated response efforts [43].

This review examines the dynamics of zoonotic disease emergence through analysis of historical pandemics and epidemics, evaluates factors driving pathogen spillover from animals to humans, and assesses preventive strategies to mitigate future pandemic risks through integrated surveillance and One Health approaches.

## 2. Methodology

To conduct this comprehensive review, we searched major scientific databases including PubMed, Web of Science, and Scopus for articles published between 1960 and 2024. The primary search terms included combinations of keywords such as “zoonotic spillover”, “cross-species transmission”, “pandemic origins”, “zoonosis prevention”, “wildlife-human interface”, “One Health approach”, and “emerging infectious diseases”. Additional searches were performed using specific pathogen names (e.g., “SARS-CoV-2”, “H5N1”, “Nipah virus”) combined with terms like “reservoir host”, “transmission”, and “prevention”. We prioritized peer-reviewed articles, systematic reviews, and meta-analyses, with particular emphasis on studies that documented spillover events, analyzed transmission mechanisms, or evaluated prevention strategies. Articles were screened for relevance based on their focus on zoonotic disease emergence, spillover mechanisms, and prevention approaches.

## 3. Historical Instances of Zoonotic Spillover Leading to Pandemics/Endemics

One of the earliest recorded instances of a devastating zoonotic spillover (Table 1) event is the Plague of Justinian, which ravaged the Byzantine Empire and Mediterranean regions in the 6th century Common Era (CE) [44]. This outbreak, caused by the bacterium *Yersinia pestis*, is believed to have originated from rodents and subsequently spread through flea vectors, resulting in widespread loss of life and economic disruption. The plague is thought to have first emerged in Egypt or Ethiopia, through trade and travel, then spread throughout the Byzantine Empire, causing massive depopulation (around to 25–100 million) in major cities, like Constantinople, profoundly impacting the social, economic, and political stability of the region.

The Black Death, a bubonic plague pandemic that swept across Europe and Asia in the 14th century, is another notorious example of a zoonotic spillover event with catastrophic consequences. This epidemic, also caused by *Yersinia pestis*, is estimated to have claimed the lives of nearly 30% of the European population [4]. The plague originated in Central Asia and spread rapidly throughout Eurasia, facilitated by the presence of infected rat and flea populations in crowded urban centers. The immense loss of life, social upheaval, and economic disruption caused by the Black Death had far-reaching implications, contributing to the decline of feudalism and the eventual transition to the Renaissance period.

Smallpox is believed to have first emerged as a human pathogen through zoonotic transmission from rodent-borne poxviruses [45]. The exact origins are unclear, but genomic studies suggest smallpox virus evolved from a virus that infected rodents in western or central Africa and subsequently crossed over to humans, potentially through human–rodent interactions. Once smallpox became established in human populations, it rapidly spread across the globe through human-to-human transmission facilitated by factors like trade, migration, conquest, and urbanization. Highly contagious and virulent, smallpox caused recurrent outbreaks and epidemics in every part of the world it reached, claiming hundreds of millions of lives over many centuries. Some of the earliest records of suspected smallpox outbreaks date back to the 3rd century Egyptian empire and 4th century China. The virus continued to devastate populations through events like the plague of Justinian and epidemics in Europe during the 18th century until it was finally eradicated through a global vaccination campaign led by the World Health Organization in the late 1970s, making it the first and only human disease to be eliminated through human efforts [46].

Several major influenza pandemics over the past century have originated from reassortment events involving the mixing of gene segments from avian, human, and/or swine influenza viruses. The catastrophic 1918 Spanish flu pandemic, which caused an estimated 50 million deaths globally, started when an avian H1N1 influenza virus acquired the ability to spread efficiently in humans [5,47]. The 1957 Asian flu pandemic resulted from the emergence of a new H2N2 influenza strain created by reassortment between avian and human viruses [48]. More recently, the 2009 H1N1 Swine flu pandemic originated from a reassortant virus that combined gene segments from swine, avian, and human influenza viruses circulating in pigs. This triple reassortant acquired the ability for sustained human-to-human transmission, rapidly spreading worldwide and replacing previously circulating seasonal H1N1 strains. While less deadly than the 1918 and 1957 pandemics, the 2009 H1N1 virus still caused over 200,000 respiratory deaths as it moved through an immunologically susceptible global population [49].

H5N1 avian influenza virus represents one of the most concerning pandemic threats due to its high pathogenicity and potential for human adaptation [50]. First identified in humans in Hong Kong in 1997, this highly pathogenic avian influenza virus primarily affects wild birds and poultry but can occasionally infect humans with devastating results. Human cases typically occur through close contact with infected birds and carry a case fatality rate exceeding 50% [51]. The virus continues to evolve, generating new variants through genetic reassortment, raising concerns about potential human-to-human transmission [52]. Recent years have seen unprecedented spread in wild bird populations globally, with mounting economic losses in poultry industries and increasing spillover events into mammals, including mink farms and sea lions, highlighting the virus’s adaptability and persistent pandemic potential [53].

Respiratory syncytial virus (RSV) is a highly contagious virus that primarily causes respiratory tract infections in humans. Evidence suggests that RSV may have originated from a genetically similar bovine respiratory syncytial virus (BRSV), which infects cattle [54] likely through close contact or exposure to infected animals or their secretions. RSV rapidly spread through person-to-person transmission, facilitated by its highly contagious nature and the ability to survive on surfaces for several hours. RSV has since become a major public health concern, particularly for infants, young children, and immunocompromised individuals, who are at higher risk for severe respiratory complications. The virus is responsible for a significant number of hospitalizations and deaths worldwide each year, highlighting the potential impact of zoonotic spillover events on human health [55].

Ebola virus is a zoonotic filovirus that causes severe hemorrhagic fever in humans and non-human primates. The origins of Ebola can be traced back to its likely reservoir hosts—fruit bats of the *Pteropodidae* family found in equatorial Africa. Ebola viruses are ancient residents in these bat populations, having evolved over millions of years to replicate asymptomatically in bat hosts. Spillover into human populations is thought to occur through direct exposure to infected bats or intermediate hosts like non-human primates that contract Ebola from bats. The first recognized outbreak of Ebola virus disease occurred in 1976 with simultaneous outbreaks in Yambuku (Zaire, now DRC) and Nzara (Sudan) [56]. The Zaire outbreak was traced back to a teacher at a mission school who was likely the index case after contracting Ebola from an unknown animal source. Person-to-person spread then occurred via close contact with infectious bodily fluids, facilitating transmission within families and healthcare settings. Since 1976, many sporadic outbreaks have occurred in equatorial Africa, but the 2014–2016 West African epidemic was the largest in history, causing over 28,000 cases as it spread through Guinea, Liberia, and Sierra Leone. With the potential for more widespread dissemination occurring through air travel of infected individuals, high mortality rate and capability of human-to-human transmission, Ebola virus represents a significant global public health threat requiring intensive surveillance and outbreak control protocols at the human–animal interface [57].

Lassa virus is a zoonotic arenavirus that causes Lassa hemorrhagic fever, an acute viral illness that can lead to bleeding, organ failure, and death in humans. The zoonotic jump from the rodent reservoir to humans occurs when people are exposed to materials contaminated with infected rodent excreta [58]. Endemic areas of Lassa fever include West African countries like Sierra Leone, Liberia, Guinea, and Nigeria. In these regions, annual seasonal outbreaks occur, likely driven by fluctuations in reservoir host populations and human agricultural activities that increase rodent–human contact. Periodic larger outbreaks have also occurred in urban centers when the virus spreads person-to-person, particularly in hospital settings, as Lassa can be transmitted through infectious bodily fluids, creating a risk of nosocomial transmission. An estimated 100,000–300,000 cases occur annually in West Africa, with the fatality rate around 1%.

Beyond its endemic regions, travel-associated cases of Lassa fever have been exported across international borders, reaching Europe, North America, and Asia [59]. This raises the potential for further geographic spread if the virus establishes transmission among non-African rodent reservoir hosts.

The severe acute respiratory syndrome (SARS) pandemic of 2003 was caused by a novel coronavirus, SARS-CoV, which likely originated in bat populations and jumped to humans in an event of zoonotic spillover. The first cases were reported in November 2002 in Guangdong Province, China, potentially stemming from exposure to live wild animals including bats, civets, and raccoon dogs at wet markets [60]. Civets are thought to have served as amplifying hosts, allowing the bat virus to adapt transiently and make the zoonotic jump to infect humans through direct contact with civets or their excreta. Person-to-person transmission of the newly emerged SARS-CoV virus then propagated the outbreak as the virus spread globally through air travel. SARS rapidly evolved into one of the most serious public health crises in recent history. Over 8000 cases occurred across 29 countries and territories before coordinated global efforts successfully contained the outbreak in 2003.

In late 2019, a novel strain of coronavirus SARS-CoV-2 began silently spreading in Wuhan, China, before triggering outbreaks across the globe. This virus caused the COVID-19 respiratory disease, which quickly overwhelmed healthcare systems and ground economies to a halt as nations implemented lockdowns to slow transmission [61]. Despite unprecedented efforts to develop and distribute vaccines at record speed, COVID-19 claimed millions of lives worldwide as vicious waves of infections swept across continents. The socioeconomic toll was immense, with businesses failing, schooling disrupted, and a mental health crises emerging. Even after case rates subsided in 2022, the long-term effects lingered in the form of long COVID, supply chain chaos, elevated debt levels, and persistent labor shortages in many sectors. The COVID-19 pandemic will be remembered as one of the most catastrophic and transformative events in modern human history [62].

Nipah virus (NiV) is a zoonotic paramyxovirus that causes severe respiratory illness and encephalitis in humans. The virus originated from bat reservoirs, the *Pteropodidae* family [31,63], and evolved from related bat-borne henipavirus lineages. The first recognized outbreak of Nipah virus disease occurred in 1998–1999 in peninsular Malaysia, where over 300 human cases were reported with over 100 deaths. This outbreak was traced back to intensively managed pig farms, where bats had access and were able to spread the virus to pigs through partially eaten fruit contaminated with saliva or urine. Pigs served as amplifying hosts, facilitating efficient transmission to pig farmers and others exposed to infected pigs or pigs’ tissues. Following this outbreak, Nipah virus was eliminated from domestic pig populations through mass culling programs. Since 1999, recurring outbreaks of human Nipah virus disease have occurred primarily in Bangladesh and surrounding regions of eastern India and are linked to consumption of date palm sap contaminated by bats [64]. Bangladesh’s 2004 NiV outbreak first provided evidence of human-to-human transmission, catalyzing heightened public health concerns. With high case fatality rates of up to 70%, Nipah represents a major threat with pandemic potential. Its geographic spread appears limited by the range of the natural bat reservoir, but environmental pressures causing bats to shift ranges could facilitate further dissemination.

West Nile virus (WNV) emerged as a significant public health threat in the Western Hemisphere when it was first detected in New York City in 1999, causing a cluster of encephalitis cases [33]. This mosquito-borne flavivirus, which naturally cycles between birds and Culex mosquitoes, has since spread rapidly across North and South America. The virus can cause severe neurological disease in humans, with approximately 1 in 150 infected people developing encephalitis or meningitis [65]. While most infected individuals remain asymptomatic, about 20% develop West Nile fever with symptoms including fever, headache, and body aches. The virus has become endemic in many regions, with annual seasonal outbreaks coinciding with mosquito activity. Climate change and urbanization have expanded suitable habitats for vector mosquitoes, potentially increasing transmission risks in new areas [66].

Rift Valley fever virus (RVFV) is a zoonotic phlebovirus that causes severe disease in both livestock and humans. First identified in Kenya’s Rift Valley in 1931, this mosquito-borne pathogen historically caused devastating outbreaks across Africa. The virus primarily affects sheep, cattle, and goats, leading to mass abortion storms in pregnant animals and high mortality in young livestock. Humans typically become infected through contact with infected animal tissues or through mosquito bites. While most human cases are mild, approximately 1% develop severe hemorrhagic fever, encephalitis, or retinal vasculitis. Major outbreaks have occurred in Egypt (1977–1978), Saudi Arabia, and Yemen (2000–2001), demonstrating the virus’s potential to spread beyond its traditional African range. The economic impact of RVFV outbreaks can be catastrophic, disrupting livestock trade and food security in affected regions.

Rabies virus, perhaps one of humanity’s longest-known viral foes, remains a significant public health threat, causing approximately 59,000 deaths annually worldwide [67]. This neurotropic lyssavirus, transmitted primarily through the bite of infected animals, has the highest case fatality rate of any viral disease, approaching 100% once clinical symptoms appear [68]. Dogs serve as the main reservoir in developing countries, while wildlife, such as bats, raccoons, and foxes, maintain transmission cycles in developed nations [68]. Despite the availability of effective pre- and post-exposure prophylaxis, rabies continues to pose a significant burden, particularly in Asia and Africa where access to medical care may be limited. The virus’s ability to infect all mammals and its lengthy incubation period make elimination challenging, though successful dog vaccination campaigns have eliminated canine rabies in many developed countries [69].

Mpox (formerly known as monkeypox) garnered global attention in 2022 when it caused unprecedented outbreaks in non-endemic countries [27]. This orthopoxvirus, first identified in laboratory monkeys in 1958 and in humans in 1970, historically caused sporadic outbreaks in Central and West Africa [36]. The virus primarily spreads through close physical contact, with symptoms including fever, distinctive skin lesions, and lymphadenopathy. While less severe than its cousin smallpox, Mpox can cause significant morbidity, with case fatality rates ranging from 1–10% depending on the viral clade. The 2022 global outbreak demonstrated the virus’s ability to establish new transmission networks in previously unaffected populations, particularly among men who have sex with men, challenging public health systems and highlighting the importance of equitable vaccine access [27].

Kyasanur forest disease virus (KFDV) and Alkhurma hemorrhagic fever virus (AHFV) are tick-borne Flaviviruses that cause hemorrhagic fevers in humans [70] and infect humans as they encroach forest habitats where reservoir hosts are present. KFDV was first identified in 1957 when it caused an outbreak of hemorrhagic fever among forest workers in the Kyasanur Forest area of Karnataka, India. Since then, the endemic zone of disease spread is expanding. Similarly, AHFV, a variant of KFDV, was first reported in 1994 following cases in the Alkhurma governorate of Saudi Arabia. This results in sporadic cases and occasional outbreaks of hemorrhagic fever near the viruses’ natural foci.

## 4. Factors Behind Zoonotic Spillover

### 4.1. Increased Human-Animal Contact

**Figure 1:**
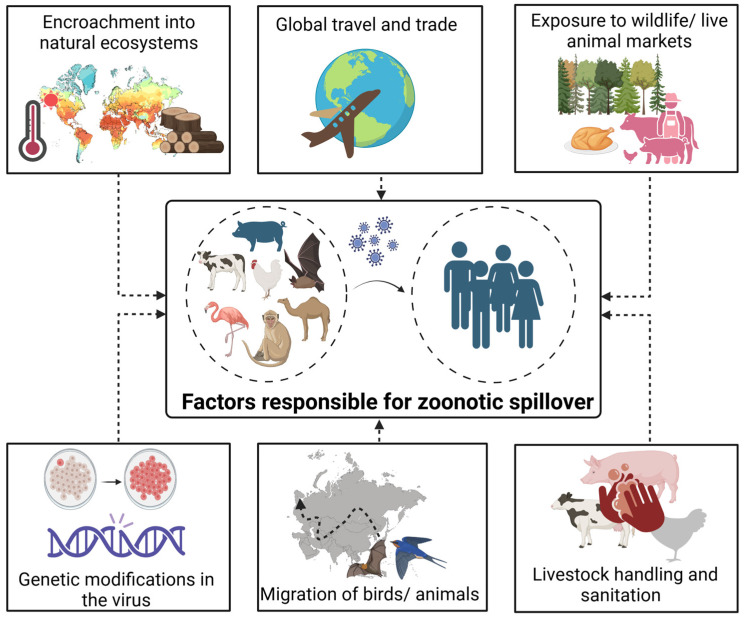
Factors underlying zoonotic spillovers.

As human populations continue to grow and expand into previously undisturbed wildlife habitats, the frequency of zoonotic spillover events escalate due to the increased opportunities for close contact between humans and animal reservoir hosts (Figure 1). This encroachment not only brings humans into direct contact with wildlife but also disrupts the delicate balance of these ecosystems, potentially increasing transmission between species, leading to the emergence of novel pathogens.

Agricultural practices, particularly intensive farming, have been closely linked to the emergence and spread of zoonotic pathogens [71]. One notable example is the outbreak of Nipah virus in Malaysia in 1998–1999. The intensive pig farming practices in close proximity to fruit orchards frequented by bats, facilitated the spillover of the virus from bats to pigs and subsequently to humans [72,73,74]. The outbreak resulted in over 100 human deaths and the culling of over a million pigs, causing significant economic losses.

Another example is the emergence of highly pathogenic avian influenza (HPAI) viruses, such as H5N1 and H7N9, which have caused numerous outbreaks in poultry farms around the world. The crowded and unsanitary conditions in industrial poultry operations, combined with inadequate biosecurity measures, create an environment conducive to the rapid spread of these viruses among birds. The close contact between poultry workers and infected birds increases the risk of zoonotic transmission, with several human cases of HPAI reported over the years [75].

In concentrated animal feeding operations (CAFOs) and industrial livestock facilities, high densities of genetically similar animals are confined in close quarters, facilitating the rapid transmission and amplification of viruses [76]. Workers in these facilities are continuously exposed to potentially infected animals, bodily fluids, and contaminated materials, increasing the chances of zoonotic spillover.

Deforestation is not just the loss of individual trees but an intricate web of ecological life dependencies that exist within those trees. When the habitat is destructed, animals are forced to seek new habitats and food sources closer to human settlements. This increased overlap between human and animal populations heightens the risk of zoonotic spillover events, as pathogens that were previously confined to wildlife reservoirs find new opportunities to jump to human hosts. For example, the Ebola virus outbreak in West Africa between 2014 and 2016, which claimed over 11,000 lives, has been associated with deforestation activities in the region [77]. When the natural habitats of fruit bats were disrupted, seeking alternative food sources brought them closer to human settlements. This contributed to the spillover of the Ebola virus from bats to the human population.

Also, migrating animals sometimes harbor vectors, such as ticks, thus increasing the risk of vectors to explore newer areas which otherwise would have sustained in the localized area. For instance, KFDV is a poignant example of a disease that has spilled over to humans due to increased encroachment for activities like cashew harvesting, highlighting the consequences of human–wildlife interactions on disease emergence [70].

Without the protective canopy, the forest floor and soil are exposed to direct rainfall, resulting in soil erosion and changes in sunlight penetration. The altered environment also promotes the growth of aquatic vegetation and algae, creating favorable conditions for mosquitoes to thrive, leading to an increase in the prevalence of mosquito-borne diseases, such as malaria, Zika, and Dengue [78]. The emergence of the Zika virus, which caused a global health crisis in 2015–2016, has been linked to deforestation in Latin America [79]. Similarly, the incidence rates of yellow fever infections have been found to be associated with deforestation trends [80].

Practices like hunting, butchering, and consuming wild animals pose direct exposure risks to humans [81]. These activities, often driven by cultural practices, traditional medicine beliefs, or food preferences, create pathways for zoonotic pathogens to jump species barriers and infect humans. Additionally, wet markets with high densities of live animals from diverse species serve as potential hotspots for the amplification, reassortment, and transmission of animal viruses to new hosts, including humans. The crowded and unsanitary conditions in these markets provide ideal environments for viruses to adapt and potentially spillover into human populations [82].

One prominent example is the HIV/AIDS pandemic, which is believed to have originated from the hunting and consumption of chimpanzees in West and Central Africa [83]. It is theorized that SIV, a precursor to HIV, was transmitted to humans through exposure to the infected chimpanzees during hunting, butchering, or the handling and consumption of bushmeat [84].

The COVID-19 pandemic, caused by the SARS-CoV-2 virus, has also been linked to the wildlife trade and the consumption of wild animals. While the exact origin is still under investigation, early cases were reported among individuals who frequented the Huanan Seafood Wholesale Market in Wuhan, China, where live wild animals were sold for human consumption [85].

### 4.2. Global Travel and Trade

The ease and frequency of international travel and the globalized trade of animals and animal-derived food products has significantly increased the potential for zoonotic viruses to rapidly disseminate across borders after an initial introduction into human populations [86].

Air travel, in particular, allows infected individuals to traverse continents in a matter of hours, potentially seeding outbreaks in new regions before symptoms even manifest. The 2003 SARS outbreak, for instance, demonstrated how a single ill traveler could spark community transmission in multiple countries within weeks [87]. Similarly, the COVID-19 pandemic spread with remarkable speed due to the high mobility of infected individuals in the early stages [88]. The unprecedented 2022 Mpox outbreak further illustrated this phenomenon, as the virus rapidly spread to over 100 non-endemic countries within months, primarily through international travel networks and close personal contact during mass gatherings. Another example is the global spread of the H1N1 swine influenza virus in 2009, which was first detected in Mexico and quickly became a pandemic [89].

The international trade of meat, poultry, and other animal-derived food items also serves as a conduit for the global movement of zoonotic pathogens if proper food safety and biosecurity measures are not followed. Contaminated food products can act as fomites, carrying pathogens into new regions, as seen with the transmission of African Swine Fever Virus through the global pig trade [90].

### 4.3. Lack of Monitoring and Surveillance

Inadequate disease monitoring and surveillance capabilities, both in human populations and animal reservoirs, significantly hinder the early detection and timely response to zoonotic spillover events. This delay in detection allows zoonotic pathogens to silently circulate and potentially establish sustained human-to-human transmission before appropriate interventions can be implemented. Furthermore, the lack of coordination and data-sharing between human and animal health sectors, often referred to as the “siloed” approach, impedes the timely recognition of zoonotic spillover events. For instance, the 2014–2016 Ebola outbreak was exacerbated by the lack of adequate surveillance systems and diagnostic capabilities in the affected countries as the initial cases went undetected for several months [91]. Similarly, during COVID-19 pandemic, many countries struggled to obtain reliable laboratory capacity to conduct widespread testing and genomic surveillance, hampering their ability to track the spread of the virus and implement targeted interventions [42].

The implementation of strategies is often hindered by a multitude of factors, including resource constraints, competing priorities, and the transient nature of human attention. Despite the availability of scientific knowledge and strategic frameworks, the lack of sustained financial investment and political commitment frequently impedes the continuity of preventive measures [92]. As in case of emergence of novel coronaviruses, while initial responses are typically mobilized, the waning of public attention and the reallocation of resources undermine the long-term sustainability of preparedness efforts [93].

### 4.4. Ecological and Climate Changes

The effects of climate change, such as rising temperatures, changing precipitation patterns, and habitat degradation, are emerging as a significant driver of alterations in the population dynamics and migratory patterns of various wildlife species, consequently creating new opportunities for zoonotic pathogens to spillover into human populations [94].

As temperatures rise, certain wildlife reservoirs may expand their ranges into new territories, bringing them into closer contact with human settlements and increasing the chances of zoonotic transmission [95]. For instance, warming temperatures in northern latitudes are allowing mosquito vectors and the pathogens they carry to establish themselves in regions previously unsuitable due to colder climates [96,97]. The establishment of West Nile virus across North America demonstrates this phenomenon, as warming temperatures have enabled Culex mosquito vectors to expand their range northward and extend their breeding season, leading to WNV detection in previously unaffected regions like Canada [55]. Similarly, the northward expansion of Aedes albopictus mosquitoes in Europe has created new opportunities for viruses like Chikungunya and Dengue to emerge in temperate regions [56]. The geographic spread of Rift Valley fever virus beyond its traditional African range into the Arabian Peninsula has also been linked to changing climatic conditions that favor mosquito vector proliferation [57]. These patterns suggest that climate change will continue to alter the distribution and transmission dynamics of vector-borne pathogens, potentially exposing immunologically naïve populations to emerging threats.

Changes in precipitation patterns, such as prolonged droughts or heavy rainfall events, can also impact the availability of food and water resources, altering the behavior and movement patterns of wildlife [98]. Moreover, climate change can influence the seasonality and lifecycle patterns of certain wildlife reservoirs and vectors, potentially extending their activity periods and enhancing their ability to transmit pathogens over longer durations or into new geographic areas [99,100].

### 4.5. Sociocultural Factors

Traditional practices like consuming bushmeat or using animal-derived traditional medicines can increase human exposure to zoonotic pathogens. As stated previously, the HIV pandemic is believed to have originated from humans hunting and consuming chimpanzee meat in central Africa. In some communities, cultural beliefs or misconceptions about disease causation and prevention may lead to resistance against modern medical interventions, including vaccines. During the 2014–2016 Ebola outbreak in West Africa, rumors that the virus was a hoax or caused by witchcraft led some villages to initially reject aid workers and healthcare measures [101].

Vaccine hesitancy fueled by misinformation or distrust in health authorities has contributed to outbreaks of vaccine-preventable diseases like measles, creating opportunities for zoonotic viruses to spread more easily. The 2019 measles outbreak in the Democratic Republic of Congo was exacerbated by low vaccination rates due to public mistrust of vaccines [102]. Additionally, hatred or stigma towards certain animal species perceived as disease carriers can hinder effective disease surveillance and control measures.

### 4.6. Inadequate Healthcare Systems

Several factors contribute to the inadequacy of healthcare infrastructure, rendering them ill-equipped to mount an effective response against rapidly spreading pathogens, exacerbating the severity and geographical reach of emerging infectious disease outbreaks [103,104].

Firstly, insufficient funding and resource allocation for public health initiatives often leave healthcare facilities understaffed, lacking essential medical supplies, and deprived of advanced diagnostic and treatment capabilities. This compromises the ability to promptly identify and isolate cases, administer appropriate care, and implement robust contact tracing measures, allowing the pathogen to propagate unimpeded. During the 2014 Ebola outbreak in West Africa, overstretched healthcare workers faced severe shortages of personal protective equipment, leading to many becoming infected themselves [105]. Secondly, limited access to healthcare services, particularly in remote and underserved areas, contributes to delayed diagnosis and treatment, facilitating sustained community transmission [104,106,107].

## 5. Challenges

### 5.1. Reservoir Diversity

One of the primary obstacles in predicting zoonotic spillover is the sheer diversity of potential viral reservoirs in the natural world. Zoonotic viruses can originate from a wide range of animal species, including bats, rodents, primates, and even domesticated livestock. For instance, bats are known reservoirs for a wide array of zoonotic viruses, such as Ebola, Marburg, Nipah, and various coronaviruses, including the progenitor of SARS-CoV-2 [108]. The ability of bats to harbor and transmit these viruses is attributed to their robust immune systems, social behaviors, and extensive geographic distribution [109].

Similarly, rodents have been implicated as reservoirs for zoonotic viruses, like hantaviruses, arenaviruses (e.g., Lassa fever virus), and certain filoviruses [110]. The ubiquitous nature of rodents in urban and rural environments, coupled with their close contact with human populations, increases the risk of viral transmission. Primates, being genetically closer to humans, can also serve as hosts for zoonotic viruses that have the potential to cross species barriers more readily [111], as evidenced by the SIVs that gave rise to HIVs.

Even domesticated livestock, such as pigs, cattle, and poultry, can harbor viruses that pose a threat to human health [112]. For example, influenza viruses circulating in swine and avian populations have the potential to reassort and generate novel strains capable of infecting humans, as seen with the 2009 H1N1 pandemic influenza virus [113].

Each of these reservoirs may harbor a multitude of viral variants, some of which may possess the ability to infect humans under the right circumstances. Identifying and monitoring these viral hotspots is a daunting task, requiring extensive surveillance efforts across vast geographical regions and often in remote or inaccessible areas. The sheer diversity of potential reservoirs, coupled with the challenges of accessing and studying these wildlife populations, makes it exceedingly difficult to comprehensively characterize the zoonotic viral threats lurking in nature.

### 5.2. Virus Adaptation and Evolution

The process of viral adaptation and evolution poses a significant hurdle in predicting zoonotic spillover events accurately. RNA viruses, in particular, are notorious for their high mutation rates and ability to rapidly evolve, allowing them to potentially acquire the necessary genetic changes to overcome species barriers and adapt to new hosts, including humans. Examples include the HIV evolving from SIVs carried by non-human primates [114]. Similarly, the influenza viruses that cause periodic pandemics are the result of reassortment events, where gene segments from human, avian, and swine influenza viruses intermix, creating novel viral strains with the ability to efficiently infect and spread among humans. Lastly, the severe acute respiratory syndrome coronavirus 2 (SARS-CoV-2) is believed to have undergone adaptive mutations that enhanced its ability to bind to human receptors and establish efficient human-to-human transmission [115].

Predicting the specific mutations or reassortment events that could confer enhanced human transmissibility, pathogenicity, or immune evasion is an immense challenge. It requires a deep understanding of viral genetics, host–pathogen interactions, and the intricate evolutionary dynamics at play. Additionally, the vast genetic diversity of viral reservoirs in animal populations, coupled with the potential for co-infections and viral recombination events, creates an enormous combinatorial space of possible evolutionary trajectories.

Furthermore, the rate and extent of viral evolution can be influenced by various factors, such as host immune pressures, antiviral treatments, and environmental conditions, adding further complexity to the predictive challenge.

### 5.3. Lack of Highly Sensitive and Robust Diagnostics

The absence of sensitive diagnostic tools and effective therapeutic interventions plays a significant role in facilitating zoonotic spillover events and enabling the subsequent spread of emerging infectious diseases. When a novel pathogen first jumps from an animal reservoir to humans, development of accurate diagnostic tests takes time, which can delay the identification and characterization of the causative agent. Meanwhile, the pathogen silently circulates and establishes sustained human-to-human transmission chains.

During 2014–2016 Ebola outbreak in West Africa, the lack of readily available diagnostic tests for Ebola virus disease (EVD) in the affected countries led to delays in recognizing the outbreak and implementing targeted interventions, allowing the virus to spread rapidly across borders [91]. Similarly, during the early phases of the COVID-19 pandemic, the limited availability of reliable SARS-CoV-2 diagnostic tests hampered efforts to track and contain the virus’s spread, contributing to its global dissemination [116,117,118].

Furthermore, the absence of effective antiviral or antimicrobial treatments can exacerbate the severity and duration of illness caused by zoonotic pathogens, increasing the potential for onward transmission [119,120].

The logistics surrounding vaccine distribution pose significant challenges. Storage requirements, such as maintaining a stringent cold chain, and ensuring long-term availability to meet global demand are critical factors that must be addressed [121,122,123].

## 6. Future Predictions

Experts warn that the coming decades could witness a surge in the number of viruses leaping between species, potentially fueling the emergence of new pandemics. A recent study published in the journal *Nature* predicts that at least 4000 instances of viruses jumping between species will occur over the next 50 years, driven by the combined effects of climate change and human encroachment on natural habitats [124]. This alarming forecast underscores the urgent need to understand the factors driving zoonotic spillover and develop effective strategies to mitigate this growing threat. The PREDICT project, funded by the United States Agency for International Development (USAID), has provided valuable insights into the scale of this challenge [125]. By surveying wildlife populations, particularly in biodiversity hotspots, the project has identified over a thousand new viruses, many of which have the potential to spill over into human populations [126]. The data represents the vast reservoir of potential zoonotic pathogens existing in the natural world. As the world grapples with the ongoing COVID-19 pandemic, the imperative to prevent the future disease spillover from animals to humans has never been more pressing.

## 7. Solutions

**Figure 2:**
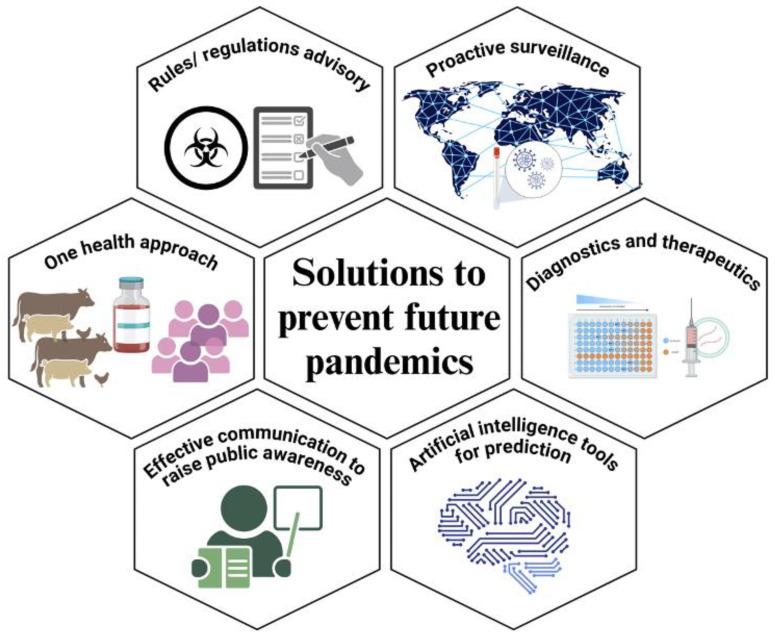
Areas to focus on for preventing pandemics.

One of the key solutions lies in strengthening surveillance and monitoring systems for zoonotic pathogens with focus on geographical locations with interactions between wild animals and humans (Figure 2). Regular sampling and testing of wildlife reservoirs, domestic animals, and vectors such as insects and ticks should be implemented to detect the presence of pathogens with zoonotic potential. Additionally, improving diagnostic capabilities and laboratory infrastructure is crucial for the rapid identification and characterization of emerging zoonotic threats. By employing advanced sequencing techniques and bioinformatics tools, researchers can comprehensively characterize the viral diversity present in various animal reservoirs and their environments [127]. Projects like the Global Virome Project aim to identify and catalogue a significant portion of the world’s viral diversity, providing a valuable resource for understanding the potential zoonotic threats lurking in nature [128]. The PREDICT project, a component of USAID’s Emerging Pandemic Threats program, has played a pivotal role in enhancing global surveillance capabilities. From 2009 to 2019, PREDICT collected over 140,000 biological samples, identified 1200 viruses with the potential to cause human disease, and trained over 6800 people in One Health surveillance in more than 30 countries. Armed with this knowledge, targeted interventions can be implemented to mitigate the spillover risk.

Advancing predictive modelling and computational approaches is crucial in forecasting zoonotic spillover events. By integrating data from various sources, including viral genomics, animal population dynamics, environmental factors, and human activities, researchers can develop sophisticated models to identify potential hotspots and assess the risks associated with specific viral threats. The Global Virome Project (GVP) initiative represent significant efforts in this direction, developing predictive models for zoonotic spillover events by analyzing the complex interplay between ecological, environmental, and socioeconomic factors [128]. These surveillance networks have been instrumental in advancing the detection and characterization of novel zoonotic viruses before they have the chance to spill over into human populations. Through proactive surveillance efforts in wildlife and domestic animal populations, initiatives like Global Health Security Agenda have identified and studied a wide range of potential pathogens with the capacity to infect humans [129,130]. By closely monitoring high-risk areas and species, these initiatives have provided early warnings of emerging threats, allowing for timely intervention and prevention measures. The emphasis on collaboration between scientists, health officials, and local communities through networks like One Health Surveillance Alliance has not only enhanced our understanding of zoonotic diseases but has also strengthened global preparedness for future pandemics [131]. These collaborative programs’ focus on rapid response and data sharing has significantly contributed to the early detection and containment of potential outbreaks, ultimately reducing the risk of spillover events and safeguarding public health on a global scale.

Sustainable wildlife management and habitat conservation play a vital role in mitigating the risk of zoonotic spillover events [132,133]. Enforcing regulations against illegal wildlife trade, hunting, and consumption of wild animals is crucial to reduce human–animal interactions that can facilitate pathogen transmission. Promoting sustainable wildlife management practices and responsible ecotourism can help maintain ecological balance and minimize human–wildlife conflicts. Protecting and restoring natural habitats is also essential to reduce the encroachment of human activities into wildlife habitats, thereby minimizing the risk of zoonotic spillover.

Implementing robust biosecurity and infection control measures is another important solution [134]. Improving biosecurity protocols in agricultural settings, such as livestock and poultry farms, can prevent the spread of pathogens between animals and humans [135]. Proper waste management and disposal systems for animal waste and by-products are essential to minimize environmental contamination and potential exposure. Promoting good hygiene practices and the use of personal protective equipment (PPE) for individuals working in high-risk settings, such as live animal markets, slaughterhouses, and veterinary facilities, can significantly reduce the risk of pathogen transmission [136].

Responsible antimicrobial use is a crucial aspect of preventing zoonotic spillover events. Promoting the judicious use of antimicrobials in animal husbandry and veterinary practices can help reduce the development of antimicrobial resistance, which can complicate the treatment of zoonotic infections [137]. Implementing guidelines and regulations for the appropriate use of antibiotics in livestock production, along with encouraging alternative strategies, like improved biosecurity, vaccination programs, and husbandry practices, can reduce the reliance on antimicrobials and mitigate the risk of resistance.

Investing in research and development is essential to address the challenges posed by zoonotic pathogens. This includes supporting research to better understand the ecology, epidemiology, and transmission dynamics of zoonotic pathogens, as well as developing rapid diagnostic tools and point-of-care tests for early detection of zoonotic diseases. Additionally, investing in the development of effective vaccines and therapeutics for both animal and human populations can contribute to prevention and control efforts.

Public awareness and education play a crucial role in preventing zoonotic spillover events. Implementing public education campaigns to raise awareness about the risks associated with human–animal interactions, wildlife trade, consumption of bushmeat and importance of taking vaccine shots on time can promote responsible personal behaviors and minimize risky practices. Promoting safe handling practices and hygiene measures when dealing with animals or animal-derived products is also important in reducing the risk of pathogen transmission.

International cooperation and collaboration are vital in addressing the global threat posed by zoonotic diseases. Fostering international cooperation and information-sharing mechanisms for early warning systems and coordinated response efforts during zoonotic disease outbreaks can facilitate timely and effective interventions. Harmonizing surveillance and monitoring protocols, as well as biosecurity standards, across countries and regions can enhance global preparedness and response capabilities. Additionally, promoting capacity-building and technology transfer to strengthen zoonotic disease prevention and control efforts in resource-limited settings is crucial for a comprehensive and equitable approach.

The successful prevention of zoonotic spillover events require a holistic and integrated strategy that addresses the complex interplay between human activities, animal reservoirs, and environmental factors. It necessitates a paradigm shift towards a “One Health” mindset, where human, animal, and environmental health are recognized as interconnected and interdependent. By fostering collaboration among experts from various disciplines, the One Health approach can inform the development of targeted interventions, such as habitat conservation efforts, stricter regulations on the wildlife trade, and the implementation of early warning systems to detect the emergence of novel zoonotic threats. By combining data on environmental changes, wildlife populations, human behavior, and disease dynamics, researchers can develop predictive models that identify high-risk interfaces and guide the allocation of resources for prevention and response efforts.

Moreover, the availability of safe and effective pre-exposure prophylactic treatments, such as vaccines or monoclonal antibodies, can provide a crucial line of defense against zoonotic threats. By proactively immunizing or conferring temporary immunity to high-risk populations, such as healthcare workers, livestock handlers, or residents in hotspot regions, we can mitigate the potential for widespread transmission and establishment of sustained human-to-human transmission chains. The successful development and deployment of the Ebola vaccine during the 2014–2016 outbreak in West Africa exemplified the potential impact of such interventions.

Complementing pre-exposure measures, the development of potent post-exposure treatments, including antivirals, antibody therapies, or novel therapeutic modalities, can significantly reduce the severity of illness and viral shedding, thereby limiting the opportunities for onward transmission. Effective treatments can also alleviate the burden on healthcare systems during outbreaks, reducing the risk of nosocomial transmission and enabling better patient management.

By investing in cutting-edge diagnostic technologies, such as point-of-care tests and genomic sequencing capabilities, as well as accelerating the research and development of innovative prophylactic and therapeutic interventions, we can enhance our preparedness and response capabilities against zoonotic threats.

Despite the daunting obstacles, the global scientific community has made significant strides in recent years, leveraging advances in fields such as genomics, computational biology, and ecological modelling to improve our understanding of zoonotic disease dynamics. Initiatives like the Global Virome Project, which aims to identify and characterize a significant portion of the world’s viral diversity, hold promise in expanding our knowledge of potential zoonotic threats. There is a need to pre-empt potential viral evolution leading to the emergence of new pathogens and development of diagnostic, prophylactic, and therapeutic tools to combat them.

## 8. Conclusions

Emerging infectious diseases are a constant threat to global health, with zoonotic spillover being a major driver. The COVID-19 pandemic has starkly highlighted the devastating consequences that can arise when a novel pathogen of animal origin gains the ability to transmit efficiently among humans. However, this is not an isolated incident; throughout history, numerous outbreaks and epidemics, such as HIV/AIDS, Ebola, influenza, and SARS, have been traced back to zoonotic origins. By understanding the complex interplay of factors that influence zoonotic spillover, such as deforestation, wildlife trade, intensive agricultural practices, and climate change, we can take proactive measures to detect and control pathogens before they cause widespread devastation.

Prioritizing preventative measures is crucial in building a more resilient public health system. Investing in robust surveillance programs that monitor zoonotic pathogens in animal populations and at the human–animal interface is essential for early detection and rapid response. Promoting sustainable wildlife management, habitat conservation, and responsible antimicrobial use in livestock production can help mitigate the risk of spillover events. However, prevention alone is not sufficient. We must also enhance our ability to rapidly develop diagnostics, vaccines, and treatments when new zoonotic threats emerge. This necessitates continued investment in research and development, fostering cross-disciplinary collaborations and strengthening global coordination and information-sharing mechanisms. By taking a proactive and comprehensive approach, addressing the underlying drivers of zoonotic spillover, and integrating scientific advancements with policy interventions and community engagement, we can better prepare for and respond to the inevitable challenges posed by emerging zoonotic diseases, safeguarding human and animal health, protecting ecosystems, and recognizing the intrinsic interconnectedness of the “One Health” concept.

## Figures and Tables

**Table 1:** Pandemics resulting from zoonotic spillovers.

Year	Disease	Pathogen	Place	Reservoir Hosts	Insect Vectors	Severity (Approximately)
1347–1353	Bubonic Plague	*Yersinia pestis*	Europe, Asia, North Africa	Rodents (primarily rats)	Fleas, human body lice	Pandemic, estimated 25–200 million deaths [10,11]
1918–1920	Spanish Flu	Influenza A virus (H1N1)	Global	Pigs	None	Pandemic, estimated 17–50 million deaths [5]
1957–1958	Asian Flu	Influenza A virus (H2N2)	Global	Ducks, geese	None	Pandemic, 1 million deaths [12]
1968–1970	Hong Kong Flu	Influenza A virus (H3N2)	Global	Pigs, birds	None	Pandemic, 1–4 million deaths [13]
2003–2004	SARS	SARS-CoV-1	China, Hong Kong, Taiwan, Canada	Bats, civets	None	8098 cases, 774 deaths [14]
2009–2010	Swine Flu	Influenza A virus (H1N1)	Global	Pigs	None	Pandemic, 151,700–575,400 deaths [15]
2012-present	MERS	MERS-CoV	Middle East, South Korea	Bats, camels	None	2499 cases, 858 deaths (as of 2019) [16]
1997-present	Avian Flu	Influenza A virus (H5N1)	Asia, Africa, Middle East, Europe	Birds	None	863 cases, 456 deaths (as of 2022) [17]
2001-present	Nipah	Nipah virus	Bangladesh, India, Malaysia	Bats, pigs	None	Outbreaks with high mortality rates [18]
2003–2004	Avian Flu	Influenza A virus (H7N7)	Netherlands	Birds	None	89 confirmed cases, 1 death [19]
2013-present	Avian Flu	Influenza A virus (H7N9)	China	Birds	None	1568 cases, 616 deaths [20]
2014–2016	Ebola	Ebola virus	West Africa	Bats, primates	None	28,616 cases, 11,310 deaths [21]
2015–2016	Zika	Zika virus	Americas, Oceania	Primates	Mosquitoes	500,000 cases estimated [22]
2019-present	COVID-19	SARS-CoV-2	Global	Bats, pangolins (suspected)	None	Ongoing pandemic, over 6.8 million deaths (as of May 2023) [23]
1999-present	West Nile	West Nile virus	Africa, Europe, Middle East, North America, West Asia	Birds	Mosquitoes	Varies by outbreak; in US: 51,607 cases, 2369 deaths (till 2019) [24]
1931-present	Rift Valley fever	Rift Valley fever virus	Africa, Middle East	Livestock (cattle, sheep, goats)	Mosquitoes	Periodic outbreaks; can cause significant livestock losses and human illness [25]
Ancient-present	Rabies	Rabies virus	Global	Mammals (dogs, bats, etc.)	None	~59,000 deaths annually worldwide [26]
1970-present	Mpox (formerly Monkeypox)	Monkeypox virus	Central and West Africa, with outbreaks globally	Rodents, primates	None	2022–2023 global outbreak: >85,000 cases, 89 deaths [27]
